# Clinical characteristics and predictors of long‐term postoperative urinary incontinence in patients treated with robot‐assisted radical prostatectomy: A propensity‐matched analysis

**DOI:** 10.1111/iju.15533

**Published:** 2024-07-17

**Authors:** Yuki Kohada, Hiroyuki Kitano, Ryo Tasaka, Shunsuke Miyamoto, Tomoya Hatayama, Hiroyuki Shikuma, Kyohsuke Iwane, Kazuma Yukihiro, Kenshiro Takemoto, Miki Naito, Kohei Kobatake, Yohei Sekino, Keisuke Goto, Akihiro Goriki, Keisuke Hieda, Nobuyuki Hinata

**Affiliations:** ^1^ Department of Urology Hiroshima University Graduate School of Biomedical Sciences Hiroshima Japan

**Keywords:** prostatectomy, prostatic neoplasms, quality of life, robot‐assisted surgery, urinary incontinence

## Abstract

**Objectives:**

This study aimed to elucidate the clinical characteristics and predictors of long‐term postoperative urinary incontinence (PUI) after robot‐assisted radical prostatectomy (RARP).

**Methods:**

This study included patients who underwent RARP at our institution and were stratified into PUI (≥1 pad/day) and continence (0 pad/day) groups at 60 months after RARP. A propensity score‐matched analysis with multiple preoperative urinary status (Expanded Prostate Cancer Index Composite urinary subdomains, total International Prostate Symptom Score (IPSS), and IPSS‐quality of life scores) was performed to match preoperative urinary status in these groups. Serial changes in urinary status and treatment satisfaction preoperatively and until 60 months after RARP were compared, and predictors of long‐term PUI were assessed using multivariate logistic regression analysis.

**Results:**

A total of 228 patients were included in the PUI and continence groups (114 patients each). Although no significant difference in preoperative urinary status was observed between the two groups, the postoperative urinary status significantly worsened overall in the PUI group than in the continence group. Treatment satisfaction was also significantly lower in the PUI group than in the continence group from 12 to 60 months postoperatively. Multivariate logistic regression analysis revealed that age (≥70 years) and biochemical recurrence (BCR) were significant predictors of the long‐term PUI group (*p* < 0.05).

**Conclusions:**

Patients with long‐term PUI had poor overall postoperative urinary status and lower treatment satisfaction than the continence group. Considering the age and risk of BCR is important for predicting long‐term PUI when performing RARP.

**Abbreviations & Acronyms**
ADT: androgen deprivation therapy
BCR: biochemical recurrence
EPIC: Expanded Prostate Cancer Index Composite
IPSS: International Prostate Symptom Score
LND: lymph node dissection
NS: nerve‐sparing
OR: odds ratio
PC: prostate cancer
PUI: postoperative urinary incontinence
QOL: quality of life
RARP: robot‐assisted radical prostatectomy
RP: radical prostatectomy
RT: radiotherapy
UB: urinary bother
UF: urinary function
UIN: urinary incontinence
UIR: urinary irritation/obstruction

## INTRODUCTION

With the availability of accessible screening methods, such as the prostate‐specific antigen (PSA) test, the diagnosis of localized prostate cancer (PC) in men has become easy.^1^ Radical prostatectomy (RP) is the gold standard treatment for localized PC, with robot‐assisted RP (RARP) being widely performed globally.^2^ RARP has demonstrated satisfactory cancer control. Therefore, increasing attention has been shifted to postoperative quality of life (QOL) and overall treatment satisfaction.^3^, ^4^

Postoperative urinary incontinence (PUI) is a major adverse event that affects patients' postoperative QOL and overall treatment satisfaction.^5^ However, only a few studies have investigated the occurrence of long‐term PUI, which was difficult to predict. Most long‐term observational studies on patients who underwent RARP have focused on PC control.^6^, ^7^ Furthermore, the assessment of postoperative urinary disorders, including PUI, is mostly based on short‐term observations of up to 1 year.^8^, ^9^ Additionally, most studies on the efficacy of interventions, such as nerve‐sparing (NS) and bladder neck preservation, which have preventive effects on PUI, are limited to short‐term observations.^5^ Identifying the characteristics and predictors of long‐term PUI will greatly contribute to improving patients' QOL after RARP.

Therefore, this study aimed to reveal the characteristics and predictors of long‐term PUI at 60 months after RARP. Serial changes in postoperative urinary status and treatment satisfaction were compared between patients with and without PUI at 60 months postoperatively after matching the preoperative urinary status between these patients by propensity score matching. Furthermore, predictors of long‐term PUI were investigated based on clinical factors and surgical procedures.

## METHODS

### Patients and study design

This study was approved by the Ethics Committee of Hiroshima University, Japan (authorization number: E2022‐0003). The medical records of 1077 patients who underwent RARP at Hiroshima University between May 2010 and September 2023 were retrospectively reviewed. The study included patients whose preoperative, 12‐month, and 60‐month follow‐up data were available for two patient‐reported outcomes measures: International Prostate Symptom Score (IPSS)^10^ and the Expanded Prostate Cancer Index Composite (EPIC).^11^ Patients with preoperative urinary incontinence (≥1 pad/day) were excluded.

### Surgical procedures

All patients in this study cohort underwent anterograde RARP via the transperitoneal approach by experienced surgeons.^12^ NS procedures were performed based on the patient's cancer risk. At our institution, we refer to four grades of posterolateral dissection of the prostate when performing NS techniques: grade 1, intrafascial dissection; grade 2, interfascial dissection; grade 3, extrafascial dissection; and grade 4, wide dissection. In this study, an NS grade of 1 or 2 on at least one side was defined as NS.^13^ We did not perform bladder neck preservation. Lymph node dissection (LND) was defined as dissection including the obturator, internal iliac nodes, and external iliac nodes.

### Assessment for urinary symptoms

All patients were invited to fill out the IPSS and EPIC questionnaires preoperatively and at 1, 3, 6, 12, 24, 36, 48, and 60 months after RARP. The IPSS comprises seven questions assessing voiding symptoms (incomplete emptying, intermittency, weak stream, and straining to void) and storage symptoms (frequency, urgency, and nocturia) and an additional QOL question assessing QOL (IPSS‐QOL).^10^ The EPIC is a validated tool to evaluate patient function and bother after PC treatment.^11^ In this study, the total IPSS, IPSS‐QOL, and EPIC urinary subdomains, namely, urinary function (UF), urinary bother (UB), urinary incontinence (UIN), and urinary irritation/obstruction (UIR), were used to assess urinary status. Treatment satisfaction was evaluated using question 32 of the EPIC questionnaire (i.e., “Overall, how satisfied are you with the treatment you received for your prostate cancer?”). Overall satisfaction was measured on a five‐point scale from 1 to 5 with 1 indicating extremely dissatisfied, 2 indicating dissatisfied, 3 indicating uncertain, 4 indicating satisfied, and 5 indicating extremely satisfied.

### Definition of continence

The number of pads used per day was collected from the medical records and was confirmed with EPIC question 5. Urinary continence was defined as using no pad, and urinary incontinence was defined as using ≥1 pad/day, including a safety pad. Patients who maintained continence at 60 months after RARP were included in the continence group, and those who experienced PUI 60 months after RARP were included in the PUI group.

### Data collection

We retrospectively collected relevant clinicopathological data, including age, body mass index, serum PSA levels, prostate volume (measured using transabdominal ultrasonography), pathological grade of prostatic biopsy (classified using the International Society of Urological Pathology grading),^14^ clinical T‐stage, National Comprehensive Cancer Network risk classification,^15^ resection margin (RM), LND, and NS. Biochemical recurrence (BCR) was defined as a PSA of ≥0.2 ng/mL.^16^ Radiotherapy (RT), androgen deprivation therapy (ADT), or combination therapy (RT + ADT) were performed for treating BCR. BCR treatment selection and timing were chosen at the physicians' discretion.

### Outcomes

Long‐term changes in urinary status and treatment satisfaction were compared between the continence and PUI groups to reveal the clinical characteristics of long‐term PUI. Predictors of long‐term PUI were investigated and assessed in detail.

### Statistical analysis

All continuous variables were presented as medians and interquartile ranges. A 1:1 propensity score matching was performed to exclude possible influences of preoperative urinary status on PUI. The propensity score was estimated using a multivariable logistic model considering covariates using preoperative UF, UB, UIR, UIN, total IPSS, and IPSS‐QOL scores. A standard caliper size (0.2 × log [SD of the propensity score]) was used. The distribution of categorical variables was compared using Pearson's Chi‐square test. If the number of events was insufficient to perform Pearson's chi‐squared test, Fisher's Exact Test was used. Differences in variables with continuous distribution across dichotomous categories were assessed using the Wilcoxon signed‐rank test. Univariate and multivariate analyses for the predictors of PUI were performed using logistic regression. All analyses were performed using R 4.3.2 (the R Foundation for Statistical Computing, Vienna, Austria). A *p*‐value <0.05 was considered statistically significant.

## RESULTS

### Patient characteristics

**FIGURE 1:**
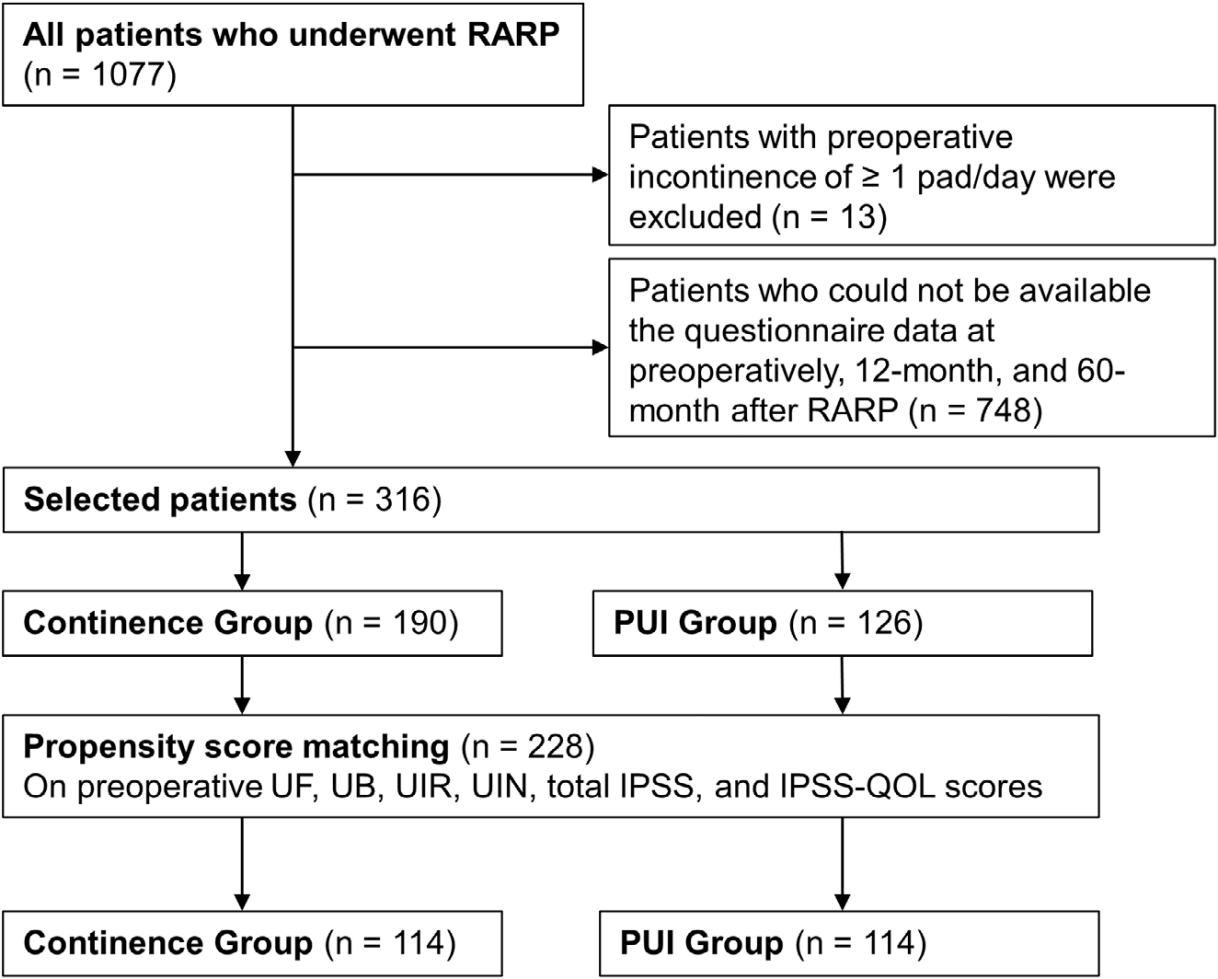
Flowchart of patient selection. IPSS, International Prostate Symptom Score; QOL, quality of life; RARP, robot‐assisted radical prostatectomy; UB, urinary bother; UF, urinary function; UIN, urinary incontinence; UIR, urinary irritation/obstruction.

A total of 316 patients were eligible for inclusion in this study. After propensity score matching was performed, 228 patients remained and were included in the continence and PUI groups (114 patients each) (Figure 1). Regarding preoperative urinary status, a significant difference in UIN was observed between the continence and PUI groups (Table 1). Significant differences in clinical features were observed between the two groups after propensity score matching (Table 1). The continence group had a higher frequency of NS (*p* = 0.009) and lower frequencies of positive RM (*p* = 0.018), LND (*p* = 0.024), and BCR (*p* < 0.001) than the PUI group. At 12 months postoperatively, 13 (11.4%) and 31 patients (27.2%) in the PUI and continence groups had 0 pad/day and had ≥1 pad/day at 12 months, respectively.

**TABLE 1:** Clinicopathological characteristics of all patients.

Variables	Overall	Before propensity score matching			After propensity score matching		
	*n* = 316	Continence Group	PUI Group	*p*‐value	Continence Group	PUI Group	*p*‐value
		*n* = 190	*n* = 126		*n* = 114	*n* = 114	
Age: median (IQR)	68 (64–72)	67 (63–71)	69 (65–73)	0.004*	67 (64–71)	69 (65–73)	0.061
Body mass index (kg/m^2^): median (IQR)	23.4 (21.7–25.1)	23.2 (21.6–24.9)	23.4 (21.8–25.5)	0.234	22.9 (21.5–24.7)	23.6 (21.8–25.5)	0.088
Initial PSA (ng/mL): median (IQR)	7.4 (5.2–11.1)	6.6 (5.1–10.5)	8.3 (5.6–11.6)	0.071	6.6 (5.1–9.6)	8.3 (5.6–11.6)	0.084
Prostate volume, (mL): median (IQR)	27 (19–36)	27 (19–38)	28 (19–35)	0.668	26 (19–35)	27 (18–35)	0.900
Pathological grade: *n* (%)				0.045*			0.111
1, 2	176 (55.7)	115 (60.5)	61 (48.4)		68 (59.6)	55 (48.2)	
3, 4, 5	140 (44.3)	75 (39.5)	65 (51.6)		46 (40.4)	59 (51.8)	
Clinical T stage: *n* (%)				0.120^a^			1.000^a^
≤cT2	309 (97.7)	188 (98.9)	121 (96.0)		112 (98.2)	111 (97.4)	
≥cT3	7 (2.3)	2 (1.1)	5 (4.0)		2 (1.8)	3 (2.6)	
NCCN risk classification: *n* (%)				0.063			0.134
Low/intermediate	230 (72.8)	146 (76.8)	84 (66.7)		89 (78.1)	78 (68.4)	
High	86 (27.2)	44 (23.2)	42 (33.3)		25 (21.9)	36 (31.6)	
Positive RM: *n* (%)	90 (28.5)	48 (25.3)	42 (33.3)	0.153	23 (20.2)	40 (35.1)	0.018*
Pelvic lymph node dissection: *n* (%)	65 (20.6)	29 (15.3)	36 (28.6)	0.006*	17 (14.9)	32 (28.1)	0.024*
Nerve sparing: *n* (%)	105 (33.2)	77 (40.5)	28 (22.2)	0.001*	44 (38.6)	25 (21.9)	0.009*
Biochemical recurrence: *n* (%)	72 (22.8)	31 (16.3)	41 (32.5)	0.001*	11 (9.6)	38 (33.3)	<0.001**
0 pad after 12 months postoperatively: *n* (%)	158 (50.0)	144 (75.8)	14 (11.1)	<0.001**	83 (72.8)	13 (11.4)	<0.001**
≥1 pad after 12 months postoperatively: *n* (%)	158 (50.0)	46 (24.2)	112 (88.9)	<0.001**	31 (27.2)	101 (88.6)	<0.001**
Pre‐IPSS total score: median (IQR)	8 (4–12)	7 (4–13)	8 (4–12)	0.523	7 (3–12)	8 (4–12)	0.415
Pre‐IPSS‐QOL score: median (IQR)	3 (2–4)	3 (2–4)	3 (2–4)	0.495	3 (2–4)	3 (2–4)	0.549
Pre‐urinary function: median (IQR)	100 (100–100)	100 (100–100)	100 (95.0–100)	0.092	100 (100–100)	100 (100–100)	0.629
Pre‐urinary bother: median (IQR)	92.9 (82.1–97.3)	92.9 (82.1–97.3)	92.9 (82.1–96.4)	0.515	92.9 (82.1–97.3)	92.9 (83.0–96.4)	0.791
Pre‐urinary incontinence: median (IQR)	100 (100–100)	100 (100–100)	100 (100–100)	0.006*	100 (100–100)	100 (100–100)	0.854
Pre‐urinary irritative: median (IQR)	92.9 (85.7–100)	92.9 (85.7–100)	92.9 (85.7–96.4)	0.542	92.9 (85.7–100)	92.9 (89.3–100)	0.783

Abbreviations: IPSS, international prostate symptom score; IQR, interquartile range; NCCN, National Comprehensive Cancer Network; PSA, prostate‐specific antigen; PUI, postoperative urinary incontinence; QOL, quality of life; RM, resection margin.

^a^ Analyzed by Fisher's exact test.

* *p* < 0.05.

** *p* < 0.001.

### Group‐wise comparison of serial changes in urinary status and treatment satisfaction after RARP

The response rates for the questionnaires at different time points were 100% (*n* = 228), 84.8% (*n* = 191), 85.5% (*n* = 195), 84.2% (*n* = 192), 100% (*n* = 228), 69.7% (*n* = 159), 73.2% (*n* = 167), 71.1% (*n* = 162), and 100% (*n* = 228) preoperatively and at 1, 3, 6, 12, 24, 36, 48, and 60 months postoperatively, respectively.

**FIGURE 2:**
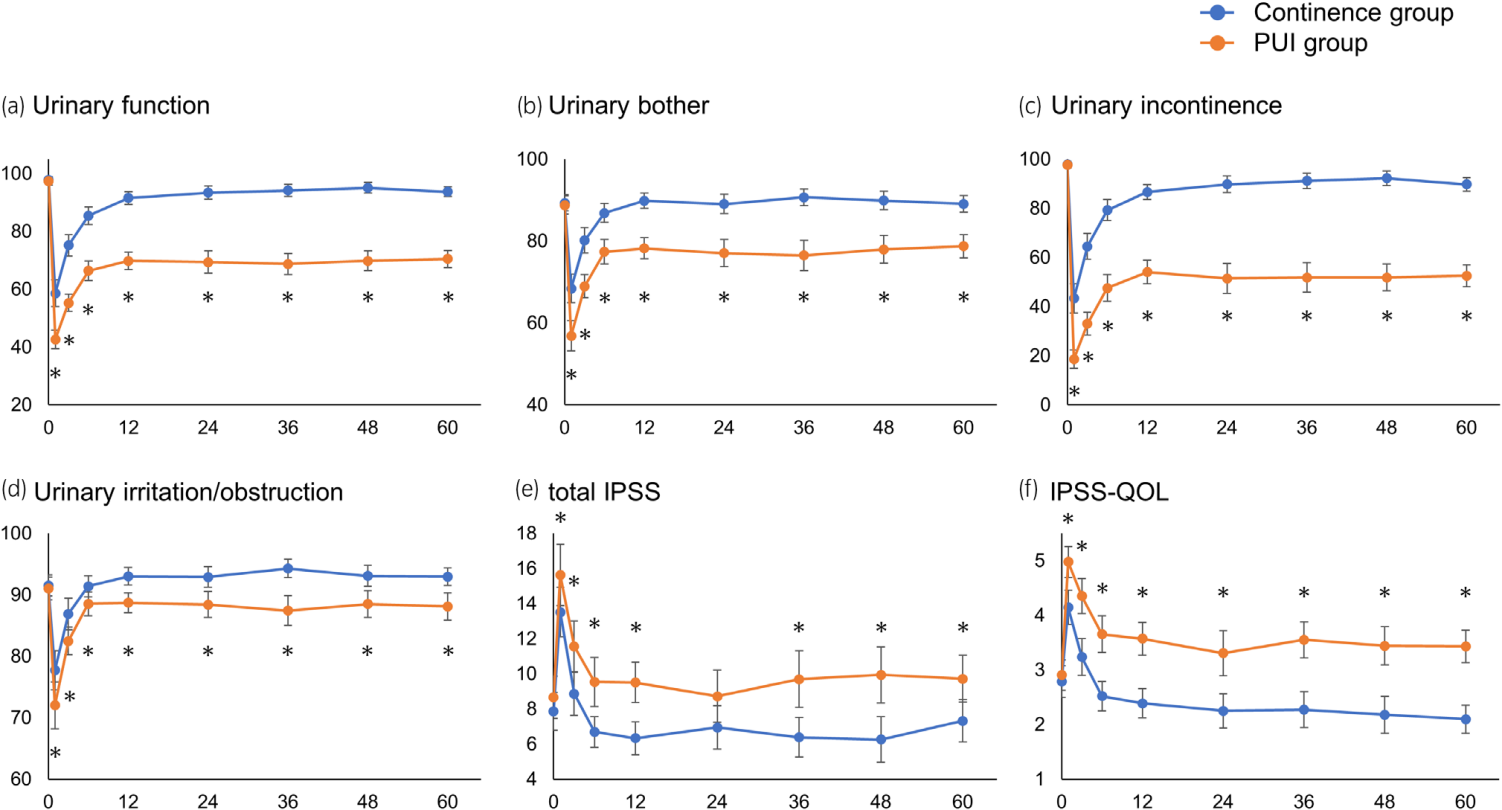
Between‐group comparison of serial changes in EPIC domain and IPSS scores. Plots represent means, and error bars represents 95% confidence intervals. Changes in (a) UF, (b) UB, (c) UIN, (d) UIR, (e) total IPSS, and (f) IPSS‐QOL scores. EPIC, Expanded Prostate Cancer Index Composite; IPSS, International Prostate Symptom Score; UF, urinary function; UB, urinary bother; UIN, urinary incontinence; UIR, urinary irritation/obstruction. *p < 0.05.

Serial changes in urinary status and treatment satisfaction after RARP were compared between the continence and PUI groups to assess differences between the two groups. In both groups, all EPIC urinary subdomain, total IPSS, and IPSS‐QOL scores worsened from preoperative levels to 1 month after RARP and gradually improved until 12 months after RARP. Then, they were maintained almost the same until 60 months after RARP (Figure 2). These findings indicate that improvements in urinary status were observed until 12 months postoperatively and could not be seen from 12 to 60 months postoperatively. Compared with the continence group, the PUI group showed worsening of the urinary status in the early postoperative period, followed by significantly fewer improvements until 12 months and significantly lower scores that were maintained until 60 months postoperatively.

**FIGURE 3:**
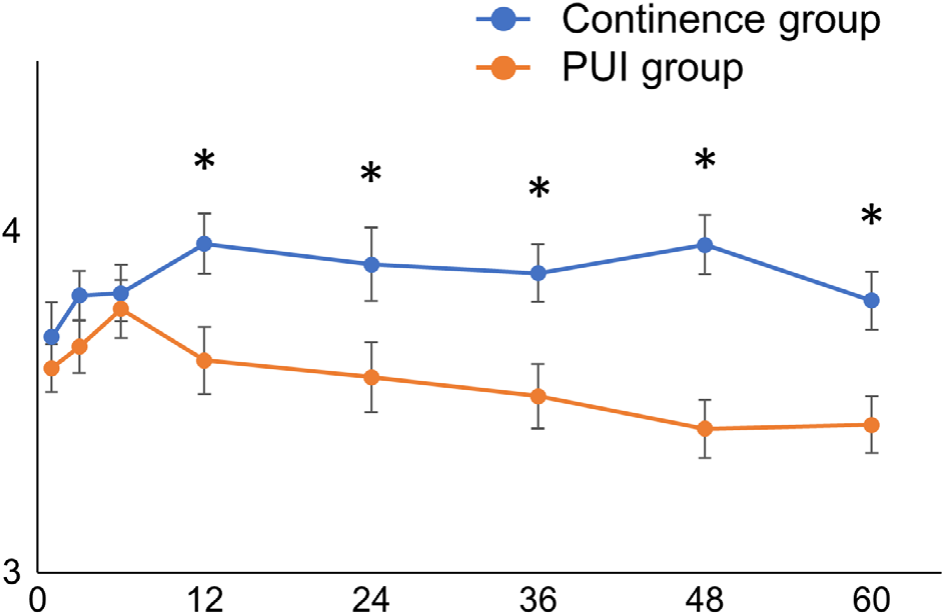
Between‐group comparison of serial changes in treatment satisfaction. Plots represent means, and error bars represents 95% confidence intervals. *p < 0.05.

Serial changes in treatment satisfaction were different from those in urinary status. Treatment satisfaction increased from 1 to 12 months postoperatively and remained almost the same in the continence group. In the PUI group, treatment satisfaction increased from 1 to 6 months, but the values remained lower than those of the continence group from 12 months to 60 months postoperatively (Figure 3).

### Assessment of predictors of long‐term PUI after RARP

Univariate and multivariate analyses were performed to determine the predictors of long‐term PUI (Table 2). Univariate analysis revealed that age ≥ 70 years (*p* = 0.022; odds ratio [OR]: 1.88), NS (*p* = 0.007; OR: 0.45), LND (*p* = 0.017; OR: 2.23), positive RM (*p* = 0.013; OR: 2.14), and BCR (*p* < 0.001; OR: 4.68) were significant predictors of long‐term PUI. Multivariate analysis revealed that age ≥ 70 years (*p* = 0.034; OR: 1.87) and BCR (*p* = 0.002; OR: 3.60) were significant predictors of long‐term PUI.

**TABLE 2:** Univariate and multivariate analyses for predictors of the PUI group.

Clinicopathological factors	Univariate analysis	Multivariate analysis
Age (≥70 years vs. < 70 years)		
OR (95%CI)	1.88 (1.10–3.25)	1.87 (1.05–3.35)
*p*‐value	0.022*	0.034*
Nerve‐sparing		
OR (95%CI)	0.45 (0.25–0.79)	0.56 (0.29–1.09)
*p*‐value	0.007*	0.088
Lymph node dissection		
OR (95%CI)	2.23 (1.17–4.38)	1.88 (0.61–5.82)
*p*‐value	0.017*	0.271
NCCN risk classification (high vs. low and intermediate)		
OR (95%CI)	1.64 (0.91–3.00)	0.51 (0.17–1.42)
*p*‐value	0.101	0.209
Positive resection margin		
OR (95%CI)	2.14 (1.19–3.93)	1.80 (0.96–3.43)
*p*‐value	0.013*	0.069
Biochemical recurrence		
OR (95%CI)	4.68 (2.32–10.16)	3.60 (1.66–8.23)
*p*‐value	<0.001**	0.002*

Abbreviations: CI, confidence interval; NCCN, National Comprehensive Cancer Network; OR, odds ratio; PUI, postoperative urinary status.

* *p* < 0.05.

** *p* < 0.001.

**FIGURE 4:**
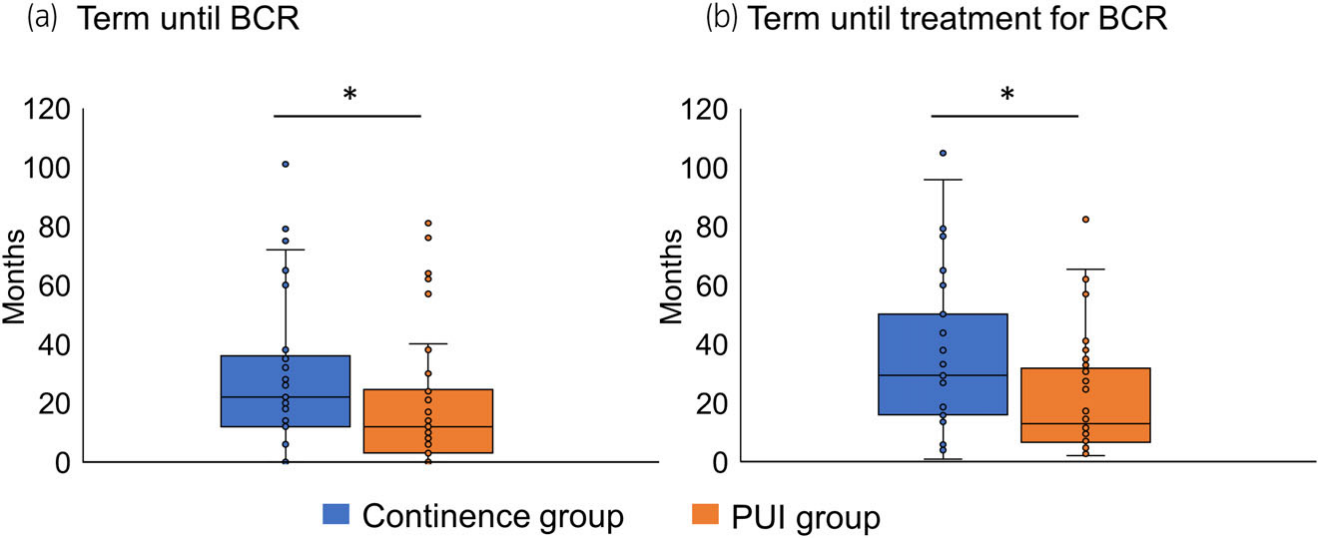
Comparison of the time until BCR (a) and BCR treatment (b) between the continence and PUI groups. BCR, biochemical recurrence; PUI, postoperative urinary incontinence. *p < 0.05.

Because BCR was revealed to be a predictor of long‐term PUI, the features of patients with BCR were compared between the continence and PUI groups. The PUI group had a significantly shorter time until both BCR and BCR treatment than the continence group (Figure 4; Table 3). Conversely, no significant difference in whether RT was included in the BCR treatment was observed between both groups (Table 3).

**TABLE 3:** Comparison of the patients with BCR between the continence and PUI groups.

	Continence Group (*n* = 11)	PUI Group (*n* = 38)	*p*‐value
Term until BCR (months): median (IQR)	23 (17–74)	13 (4–25)	0.019*
Term until treatment for BCR (months): median (IQR)	29 (22–77)	14 (9–32)	0.007*
Treatment of BCR			0.706
RT or RT + ADT (%)	7 (63.6)	28 (73.7)	
ADT (%)	4 (36.4)	10 (26.3)	

Abbreviations: ADT, androgen deprivation therapy; BCR, biochemical recurrence; IQR, interquartile range; PUI, postoperative urinary incontinence; RT, radiotherapy.

* *p* < 0.05.

Finally, we evaluated urinary incontinence status at 60 months after RARP in patients using 0 and ≥1 pad/day at 12 months postoperatively (Table 4). We found that 31 (23.5%) patients using ≥1 pad/day at 12 months postoperatively improved to the continence group at 60 months. Furthermore, those who achieved 0 pad/day from 12 to 60 months had significantly lower age and BCR rates compared with those who did not. In contrast, 13 (13.5%) patients using 0 pad/day at 12 months postoperatively worsened to the PUI group at 60 months, and they had significantly higher BCR rates than those who did not.

**TABLE 4:** Evaluation of urinary incontinence status at 60 months after RARP and comparison of clinicopathological factors in patients using 0 and ≥1 pad/day at 12 months postoperatively.

Variables	0 pad/day after 12 months postoperatively: *n* (%)			≥1 pad/day after 12 months postoperatively: *n* (%)		
	Continence Group	PUI Group	*p*‐value	Continence Group	PUI Group	*p*‐value
	*n* = 83 (86.5)	*n* = 13 (13.5)		*n* = 31 (23.5)	*n* = 101 (76.5)	
Age: median (IQR)	69 (62–73)	67 (64–71)	0.983	66 (62–69)	69 (65–73)	0.021*
Body mass index (kg/m^2^): median (IQR)	22.6 (21.1–24.4)	22.7 (20.1–24.9)	0.953	23.7 (22.2–25.3)	23.6 (22.0–25.8)	0.857
Initial PSA (ng/mL): median (IQR)	6.7 (5.1–9.7)	7.5 (7.1–9.6)	0.214	6.0 (5.1–9.6)	8.4 (5.3–11.6)	0.735
Prostate volume, (mL): median (IQR)	26 (20–37)	19 (15–32)	0.346	28 (18–35)	28 (20–35)	0.188
Pathological grade: *n* (%)			0.367^a^			0.299^a^
1, 2	51 (61.4)	6 (46.3)		19 (61.3)	49 (48.5)	
3, 4, 5	32 (38.6)	7 (53.7)		12 (38.7)	52 (51.5)	
Clinical T stage: *n* (%)			1.000^a^			1.000^a^
≤cT2	82 (98.8)	13 (100.0)		30 (96.8)	98 (97.0)	
≥cT3	1 (1.2)	0 (0.0)		1 (3.2)	3 (3.0)	
NCCN risk classification: *n* (%)			1.000^a^			0.398^a^
Low/intermediate	65 (78.3)	10 (76.9)		24 (77.4)	68 (67.3)	
High	18 (21.7)	3 (23.1)		7 (22.6)	33 (32.7)	
Positive RM: *n* (%)	17 (20.5)	5 (38.5)	0.167^a^	6 (19.4)	35 (34.7)	0.165^a^
Pelvic lymph node dissection: *n* (%)	11 (13.3)	2 (15.4)	1.000^a^	6 (19.4)	30 (29.7)	0.368^a^
Nerve sparing: *n* (%)	34 (41.0)	5 (38.5)	1.000^a^	10 (32.3)	20 (19.8)	0.229^a^
Biochemical recurrence: *n* (%)	10 (12.0)	7 (53.7)	0.001^a^, *	2 (6.5)	31 (30.7)	0.013^a^, *

Abbreviations: IQR, interquartile range; NCCN, National Comprehensive Cancer Network; PSA, prostate‐specific antigen; PUI, postoperative urinary incontinence; RM, resection margin.

^a^ Analyzed by Fisher's Exact Test.

* *p* < 0.05.

## DISCUSSION

The novelty of this study is that it attempted to match preoperative urinary status when comparing the continence and PUI groups and defined “No pad” as long‐term PUI after RARP. Although the appropriate method of clinical evaluation for urinary continence after RARP remains controversial, the “No pad” criterion is reported to be the least biased and fairest option.^17^ Conversely, despite scattered reports on long‐term PUI after RARP, none have addressed differences in preoperative urinary status.^18^, ^19^ Poor preoperative urinary status can predict PUI after RARP,^18^ and which perioperative factors affecting long‐term urinary incontinence in patients with the same preoperative background regarding urinary status are still controversial. Matching the preoperative background regarding urinary status before comparing the continence and PUI groups is crucial to assess the impact of clinicopathological factors and therapeutic interventions on long‐term PUI after RARP.

In this study, a comparison between the continence and PUI groups was made in terms of urinary status and treatment satisfaction from preoperative level to 60 months after RARP. Although similar preoperative urinary status was observed between the two groups, the postoperative urinary status significantly worsened overall in the PUI group. Although improvement was observed in the PUI group up to 12 months, this improvement was insufficient, and the status remained low and unchanged after that. It has already been reported that it is not easy to obtain improvement in urinary status after 12 months postoperatively.^18^, ^19^ Conversely, we found that some patients using ≥1 pad/day at 12 months postoperatively improved to the continence group at 60 months. The same result has been reported, and assessing mean functional scores or recovery rates of all men from the time of their RP onward may not appropriately evaluate a particular population, such as patients with improved urinary continence from 12 to 60 months postoperatively.^20^ These findings indicate that interventions contributing to improved PUI, such as pelvic floor muscle training, should be implemented and performed as early as possible and continued long‐term postoperatively.^5^ Interestingly, this study showed that treatment satisfaction in the PUI group was significantly lower than that in the continence group from 12 months to 60 months postoperatively. Previous studies have reported treatment satisfaction tends to decrease as the observation period lengthens.^21^ Our findings indicate that this trend might be stronger in the PUI group. Thus, ongoing management for improving urinary incontinence during follow‐up may be important to maintain treatment satisfaction. Treatment satisfaction has been reported to be influenced by various factors other than urinary status, such as erectile dysfunction and tumor recurrence.^21^, ^22^ Thus, further studies are needed to investigate the influence of these factors.

This study showed that old age (≥70 years) and BCR were predictive factors for PUI 60 months after RARP. Older age (≥70 years) has been reported to be a cause of long‐term PUI.^23^, ^24^ Thus, considering the patient's age at the time of surgery is important when treating localized PC. Additionally, although the association between BCR and long‐term PUI has never been reported, postoperative RT (salvage/adjuvant) and ADT have been reported to be associated with the worsening of PUI.^25^, ^26^ Although no difference was observed in the selected treatment, whether containing RT or not, between the two groups, the time until BCR and BCR treatment was shorter in the PUI group than in the continence group. Despite various opinions, early postoperative treatment of BCR has been reported to be associated with the worsening of PUI.^27^, ^28^ In this study, the results showed that both the time until BCR and BCR treatment in the PUI group generally coincided with the duration of improvement in urinary status after RARP, whereas those in the continence group did not. This finding indicates that early BCR and subsequent treatment, including the postoperative period of improvement in urinary status, may have affected long‐term PUI. However, further studies are needed to determine the extent to which the time until BCR and BCR treatment strongly affects postoperative urinary status.

The results of this study provide valuable insights into the prevention and management of long‐term PUI after RARP. It is important to provide patients with appropriate information in informed consent, especially patients with ≥70 years old or those with high‐risk localized PC patients who are predicted to have BCR at an early postoperative period, to improve their treatment satisfaction. Furthermore, efforts to understand the anatomy around the prostate to perform radical surgery that reduces the risk of BCR are important.^29^ Surgical interventions for PUI have been reported to contribute to improving patients' QOL.^30^ Thus, patients who continue to suffer from PUI after 12 months postoperatively should be actively considered for surgical interventions, such as the use of an artificial urinary sphincter.

This study has several limitations. This was a single‐center study with a small sample size. Particularly, we included a small sample size of patients with BCR. The questionnaire's low response rates at 1, 3, 6, 24, 36, and 48 months postoperatively may have influenced the results. Furthermore, the standardized strategy for selecting of the treatment for BCR and the timing when the treatment started were not adopted in this study. The EPIC urinary subdomain and IPSS were used as factors for propensity score matching. However, because there are other evaluations of urinary status, such as the overactive bladder symptom score, perfect matching of preoperative urinary status might not be achieved. Because we cannot detect patients who will develop BCR postoperatively during surgery, the predictive ability of BCR for long‐term PUI may be considered less important than that of other perioperative factors. However, because some patients showed improved urinary incontinence status after 12 months postoperatively, early BCR is valuable for predicting long‐term PUI, particularly in these patients (Table 4). Despite these limitations, the findings of this study can help physicians make informed treatment decisions for patients with localized PC. A future prospective multicenter study is needed to verify the present results.

In conclusion, the PUI group showed insufficient improvement regarding various urinary status, maintained a poor urinary status for a long‐term after RARP, and tended to have lower treatment satisfaction than the continence group. Age and BCR, particularly early recurrence after surgery, are predictive factors for long‐term PUI. These findings help physicians make informed treatment decisions for localized PC and long‐term management of PUI.

## AUTHOR CONTRIBUTIONS

**Yuki Kohada:** Conceptualization; data curation; writing – original draft; project administration; visualization. **Hiroyuki Kitano:** Visualization; writing – review and editing. **Ryo Tasaka:** Data curation; writing – review and editing. **Shunsuke Miyamoto:** Data curation. **Tomoya Hatayama:** Data curation. **Hiroyuki Shikuma:** Data curation. **Kyohsuke Iwane:** Data curation. **Kazuma Yukihiro:** Data curation. **Kenshiro Takemoto:** Data curation. **Miki Naito:** Data curation. **Kohei Kobatake:** Data curation. **Yohei Sekino:** Data curation. **Keisuke Goto:** Data curation. **Akihiro Goriki:** Data curation. **Keisuke Hieda:** Data curation. **Nobuyuki Hinata:** Supervision.

## CONFLICT OF INTEREST STATEMENT

Nobuyuki Hinata is an Editorial Board member of International Journal of Urology and a co‐author of this article. To minimize bias, they were excluded from all editorial decision‐making related to the acceptance of this article for publication.

## APPROVAL OF THE RESEARCH PROTOCOL BY AN INSTITUTIONAL REVIEWER BOARD

Institutional review board, ethics committee or ethical review board study approval: number is E2022‐0003.

## INFORMED CONSENT

All human subjects provided written informed consent with guarantees of confidentiality.

## REGISTRY AND THE REGISTRATION NO. OF THE STUDY/TRIAL

N/A.

## ANIMAL STUDIES

Institutional animal care and use committee approval: N/A.
